# Knowledge of prevention of mother-to-child transmission of HIV among reproductive age women in high HIV/AIDS prevalent countries: A multilevel analysis of recent Demographic and Health Surveys

**DOI:** 10.1371/journal.pone.0292885

**Published:** 2023-10-12

**Authors:** Habitu Birhan Eshetu, Natnael Kebede, Eyob Ketema Bogale, Amare Zewdie, Tadele Derbew kassie, Tadele Fentabil Anagaw, Elyas Melaku Mazengia, Sintayehu Shiferaw Gelaw, Eneyew Talie Fenta

**Affiliations:** 1 Department of Health Promotion and Health Behaviour, Institute of Public Health, College of Medicine and Health Sciences, University of Gondar, Gondar, Ethiopia; 2 Department of Health Promotion, School of Public Health, College of Medicine and Health Sciences, Wollo University, Dessie, Ethiopia; 3 Department of Health Promotion and Behavioral Science, School of Public Health, College of Medicine and Health Science, Bahir Dar University, Bahir Dar, Ethiopia; 4 Department of Public Health, College of Medicine and Health Science, Wolkite University, Wolkite, Ethiopia; 5 Department of Public Health, College of Medicine and Health Science, Debre Markos University, Debre Markos, Ethiopia; 6 Department of Public Health, College of Medicine and Health Sciences, Injibara University, Injibara, Ethiopia; Boston University School of Medicine, UNITED STATES

## Abstract

**Background:**

A lack of enough knowledge about the mother-to-child transmission (MTCT) of the human immunodeficiency virus (HIV) among reproductive-age women is thought to be a key contributor to new pediatric HIV infections worldwide, and rising HIV-related infant mortality, particularly in resource-limited countries. Knowledge of MTCT of HIV is key to halt the progression of HIV/AIDS. Therefore, this study aimed to assess knowledge of MTCT of HIV and its associated factors among reproductive-age women in high HIV/AIDS prevalent countries.

**Methods:**

A secondary data analysis was performed using 8 HIV/AIDS prevalent countries’ Demographic and Health Surveys. A total weighted sample of 97,130 respondents was included in this study. Stata 17 was used for data extraction, coding, and analysis. A multilevel binary logistic regression model was fitted. The odds ratios along with the 95% confidence interval were generated to determine the factors of good knowledge of MTCT of HIV among reproductive-age women. A 95% confidence interval and a p-value of less than 0.05 were used to declare statistical significance.

**Results:**

The prevalence of good knowledge of MTCT HIV was 57.89% (95% CI: 57.67,58.29). Mothers aged 25–34 (AOR  =  1.37, 95% CI = 1.32, 1.42), 35 and above(AOR  =  2.46,95% CI = 1.41, 1.52), mothers’ primary education (AOR  = 1.32, 95% CI = 1.26, 1.38),), secondary education (AOR  = 1.65,95% CI = 1.56, 1.74), higher education (AOR  = 1.72,95% CI = 1.58,1.86), exposed to mass media (AOR  = 1.12, 95% CI = 1.08,1.16) rich wealth status (AOR  = 1.11 (95% CI = 1.06,1.15), talked about MTCT (AOR  = 1.70,95% CI = 1.64, 1.76), visited by field worker (AOR  = 1.09, 95% CI = 1.03,1.14), health facility visit (AOR  = 1.15, 95% CI = 1.11, 1.18), urban dwellers (AOR = 1.09, 95% CI = 1.04,1.14), ever tested for HIV(AOR = 2.18 (95% CI = 2.10,2.27), currently working status (AOR = 1.15, 95% CI = 1.12,1.19) were factors associated with good knowledge of MTCT of HIV/AIDS among reproductive age women.

**Conclusions:**

Overall, the prevalence of good knowledge of MTCT was low in high HIV/AIDS prevalent countries. Maternal age, primary education and above, exposed to media, having higher wealth status, talked about MTCT during ANC visits, being visited by a field worker, visited a health facility, currently working, living in the urban area, and ever been tested for HIV were positively associated with knowledge of MTCT. Health policy and programs should focus on educating mothers, encouraging women to contact health facilities and a well-targeted communications program is required to enhance knowledge of MTCT of HIV.

## Background

Globally, 1.5 million people were newly infected with HIV in 2021, and about 40.1 million people were living with HIV in 2023 [1, 2]. Of these, two-thirds (25.6 million) are in the world health organization (WHO) African Region [3, 4]. HIV/AIDS remains the leading cause of death in sub-Saharan Africa [5]. In order to combat the HIV pandemic, the Joint United Nations Programme on HIV/AIDS (UNAIDS) created the Fast-Track Strategy in 2014, which targets low- and middle-income countries in order to accomplish sustainable developmental goal (SDG) 3.3 (End AIDS by 2030) [6]. Even though different efforts were being made, in 2021, the global burden of pediatric HIV infection was 1.7 million children under the age of 15, with 98 000 deaths as a result [2].

Mother-to-child transmission (MTCT), also known as vertical HIV transmission, has been identified as the primary cause of HIV infection in children under the age of 15 years [1]. MTCT happens when an HIV-positive mother transmits the virus to her kid during the course of pregnancy, labour, delivery, or nursing [3, 7]. It is believed that MTCT causes more than 90% of all HIV infections in children [8, 9], and is highest in Sub-Saharan African (SSA) region [10].

Among the top prevalent countries in the world, the prevalence of HIV/AIDS ranges from 7.3% to 26.8% in Equatorial Guinea and Eswatini, respectively in 2020 [11]. To reduce the prevalence of HIV/AIDS, the WHO recommends reducing MTCT through the prevention of HIV among women of reproductive age, preventing unplanned pregnancies among HIV-positive women, and providing antiretroviral therapy (ARTs) to HIV-positive mothers [3, 12]. Thanks to HIV prevention programs, it has been 1.5 million deaths and 2.9 million HIV infections averted among pregnant women and children since the beginning of mother-to-child transmission prevention programs [13].

In order to eliminate MTCT of HIV, maternal knowledge of MTCT and its prevention is a cornerstone. Besides, it is a path to reach the UNAIDS 95:95:95 targets [i.e., 95% of people living with HIV are aware of their diagnosis, 95% receive ART, and 95% have sustained viral suppression on ART] [14, 15], and 2030 target of ending AIDS as a public health threat as defined in the SDGs [16]. However, evidence have suggested that, despite broad information, education, and communication initiatives, as well as the expansion of PMTCT services, women’s knowledge of risk factors for MTCT of HIV and times of transmission is severely limited [17, 18]. Knowledge of MTCT specifically varies from country to country as well as from the study population, for instance, knowledge of MTCT of HIV among pregnant women in Ethiopia showed 34.9% [9], and 70.5% in Zimbabwe [19]. According to different studies conducted, knowledge of MTCT and PMTCT of HIV/AIDS was associated with factors such as maternal age, residence, maternal education, occupation of mother, wealth status, marital status, and media exposure [3, 9, 19, 20], HIV/AIDS status [21], and prior utilization of PMTCT services [22].

Correct knowledge of MTCT and PMTCT among reproductive-age women is critical because it influences behavior modification and supports the adoption of self-protective attitudes such as increased perceived vulnerability, condom usage, and HIV testing [23, 24]. Knowledge of MTCT and PMTCT of HIV and the factors that hinder or facilitate it is a significant aspect of study among reproductive-age women, to enhance the utilization of PMTCT services. Moreover, it has not been studied in the setting of high HIV-burden countries. Therefore, we aimed to assess the level of knowledge regarding mother-to-child transmission (MTCT) of HIV/AIDS and identify the factors associated with this knowledge among women of reproductive age in countries with a high burden of HIV/AIDS. To achieve this, we utilized the most recent data from the Demographic and Health Surveys (DHS) conducted in the respective countries. By employing rigorous scientific methods, we aimed to provide accurate and reliable insights into the subject matter. It is hoped that the results of the study will help policymakers to make interventions that will help increase awareness of MTCT in return reduce MTCT and related morbidity by speeding up their awareness among high HIV prevalence countries.

## Methods

### Data source and study population

In this study, we have used the top ten HIV/AIDS prevalent countries’ DHS, conducted from 2011 to 2018. Among those countries, there were eight countries DHS conducted in the study period. Eswatini and Botswana were excluded because of the absence of DHS data. Those countries with the Highest HIV Rates by increasing order include (Equatorial Guinea, Malawi, Zambia, Mozambique, Namibia, Zimbabwe, South Africa, Botswana, Lesotho, and Eswatini) [11]. The data for these eight top ten HIV-prevalent countries were gathered from the Demography Health Survey (DHS) program’s official database, www.measuredhs.com after authorization was given by online request by stating the objective of our study on June 07/2023. We retrieved the outcome and independent variables from the woman record (IR file) data set. DHS is a nationally representative household survey that employs face-to-face interviews to follow and analyze a wide range of population, health, and nutrition parameters. The stratified, multi-stage, random sampling approach was used in the surveys. Detailed survey procedures and data collection sample methods have been reported somewhere [24]. For this study, a total weighted sample of 97,130 reproductive-age women was included.

### Study variables

#### Dependent variable

The dependent variable in this study was knowledge of MTCT of HIV/AIDS. It was a composite score of four questions according to the DHS Guideline: “Know that HIV can be transmitted from mother to child during pregnancy (yes/no)”, “know that HIV can be transmitted from mother to child during delivery (yes/no)”, “know that HIV can be transmitted from mother to child by breastfeeding (yes/no) “, and “know that the risk of mother-to-child transmission can be reduced by the mother taking special drugs(yes/no)” [25]. Then a woman had good knowledge of MTCT of HIV if all the four questions are answered correctly (if a women said yes to all four questions), otherwise considered not knowledgeable.

#### Independent variables

In this study, we have used individual and community level variables to assess factors associated with knowledge of MTCT of HIV/AIDS among reproductive-age women in high HIV prevalent countries.

*Individual-level variables*. Maternal age, maternal education, current working status, current marital status, household wealth status, mass media exposure, visited the health facility, visited by field worker, during antenatal visit talked about mother to child HIV transmission, and Ever been tested for HIV were incorporated as individual-level factors.

*Community-level variables*. Residence, community level women’s literacy, community level poverty, community level of media exposure, distance from the health facility, and country were the community-level variables. The factors like residence (i.e. urban/rural) and country are as collected by the DHS survey. For the rest of the community level variables, the individual level responses were aggregated and categorized based on the score of each variable. community-level media exposure was created by adding together all the "yes" responses in each area from women’s exposure to radio, newspaper/magazine, and television. Since the distribution of the variables were not normal based on subjective tests like histogram, we have used the median as a cut of point to categorize as low and high. When the amount of media exposure exceeded or was equal to the median, it was classified as high; otherwise, it was classified as low. The concept of community-level women’s literacy was developed by classifying women with no formal education as "no" and those with primary, secondary, and higher education as "yes". The proportion was then determined from the "yes" education group and categorized as "0" for low (communities in which less than 50% of women had at least a primary education) and "1" for high (clusters in which > = 50% of women had at least a primary education).

### Data management and analysis

Stata version 17 software was used to extract, recode, and for the analysis. The DHS data is hierarchical so we have used multilevel analysis. To keep the representativeness, to take into account the non-response rate, and to get an appropriate statistical estimate (robust standard error) weighting was applied throughout the analysis [26]. In this analysis, we have fitted four models. The first model (empty model) was fitted in the absence of any independent variables to assess the variability of the outcome between clusters. Model 2 which was done by including individual-level variables alone. Model 3 community level variable only and the fourth (Model 4) models were fitted with both individual and community level variables. For random effect analysis, to evaluate the cluster level variance of knowledge of MTCT, ICC and proportional change in variance (PCV) were used. Model fitness was checked by deviance, and the best-fit model was a model with the lowest deviance. In our analysis, the ICC was found to be less than 5% it suggests that there is relatively little variation in the outcome variable between higher-level units (i.e between clusters) compared to the total variation. In other words, most of the variation in knowledge of MTCT of HIV is at the individual level rather than between the clusters. However, we have conducted the multilevel analysis due to the hierarchical nature of the DHS data.

To find relevant variables for the multivariable analysis, a bivariable analysis was done. Variables having p-values less than or equal to 0.25 in the bivariable analysis were used for the multivariable analysis. In the multivariable analysis, the adjusted odds ratio (AOR) with its 95% confidence interval (CI) was presented, and variables with p-value less than or equal to 0.05 were judged significant factors of knowledge of MTCT of HIV/AIDS.

### Ethical consideration

Ethics approval was not required for this study since the data is secondary and is available publicly. However, we have been given the authorization letter to download the DHS data. More details concerning DHS data and ethical standards are available at http://goo.gl/ny8T6X.

## Results

### Sociodemographic characteristics of the respondents

This study examined a total weighted sample of 97,130 reproductive women using the recent DHS dataset. The mean age was (M = 28.57 years, SD = 9.54), and the majority (40.39%) of respondents were between the ages of 15 and 24. The vast majority of participants (62.01%) lived in rural regions. About 25.29% of the respondents were from Malawi, whereas Lesotho, a landlocked kingdom, had the fewest (6.82%) survey participants. Only a small number (5.51%) of the respondents had higher education. Furthermore, over 44% were identified in the rich wealth quantiles, while over 36% were found in the poor wealth quantiles. Seventy-one percent of the respondents had ever been tested for HIV (Table 1).

**Table 1:** Socio-demographic and other characteristics of respondents in high HIV prevalent countries of SSA, N = (97,130).

Variables	Category	Frequency	Percent
Age of respondents in years	15–24	39226	40.39
	25–34	30191	31.08
	≥35	27713	28.53
Residence	Urban	36903	37.99
	Rural	60227	62.01
Country	Equatorial Guinea	10874	11.2
	Lesotho	6621	6.82
	Malawi	24562	25.29
	Mozambique	13745	14.15
	Namibia	9176	9.45
	South Africa	8514	8.77
	Zambia	13683	14.09
	Zimbabwe	9955	10.25
Sex of household head	Male	62990	64.85
	Female	34140	35.15
Current marital status of respondents	Married	47768	49.18
	Not married	49362	50.82
Educational status of respondents	No education	16593	17.08
	Primary education	37162	38.26
	Secondary education	38020	39.14
	Higher education	5356	5.51
Educational status of partner(n = 91559)	No education	13231	22.07
	Primary education	21918	36.56
	Secondary education	20601	34.36
	Higher education	4208	7.02
Media exposed	No	28,139	28.97
	Yes	68,991	71.03
Respondents currently working	No	49541	51
	Yes	47589	49
Wealth status	Poor	35410	36.46
	Middle	18338	18.88
	Rich	43382	44.66
Visit the health facility for the last 12 months	No	41638	42.87
	Yes	55492	57.13
Distance to the health facility	Big problem	38594	39.73
	Not big problem	58536	60.27
Visited by fieldworker in last 12 months	No	87484	90.07
	Yes	9646	9.93
Ever been tested for HIV	No	27756	28.58
	Yes	69374	71.42
Know a place to get HIV test	No	12550	12.92
	Yes	84580	87.08
During antenatal visit talked about: HIV transmitted mother to child	No	71653	73.77
	Yes	25477	26.23
During antenatal visit talked about: things to do to prevent getting HIV	No	71683	73.8
	Yes	25447	26.2
During antenatal visit talked about: getting tested for HIV	No	70591	72.68
	Yes	26539	27.32
Tested for HIV as part of antenatal Visit	No	70192	72.27
	Yes	26938	27.73
Currently pregnant	No	89805	92.46
	Yes	7325	7.54
A decision about Health care	respondent alone	15305	15.76
	respondent and husband/partner	25211	25.96
	husband/partner alone	15777	16.24
	someone else	439	0.45
	Missing	40398	41.59
Community media exposure	Low	48358	49.79
	High	48772	50.21
Community poverty level	Low	48970	50.42
	High	48160	49.58
Community women’s literacy	Low	48551	49.99
	High	48579	50.01

### Reproductive age women’s knowledge of MTCT of HIV

**Fig 1:**
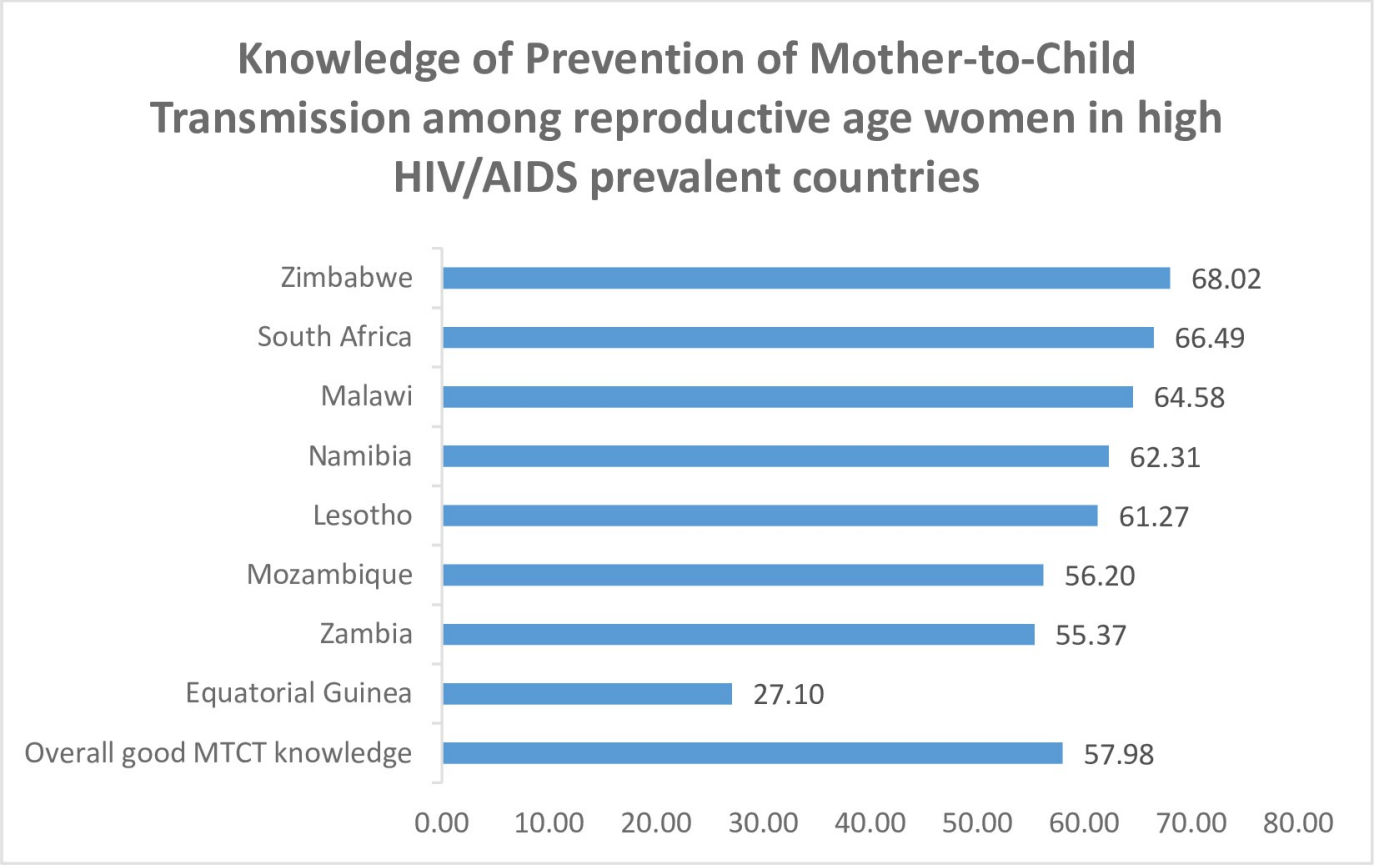
Knowledge of prevention of mother-to-child transmission among reproductive age women in high HIV/AIDS prevalent countries.

The percentage of reproductive-age women’s knowledge on MTCT revealed 57.89% (95% CI: 57.67,58.29). The lowest and the highest Knowledge of MTCT was observed in Equatorial Guinea and Zimbabwe 27.10% and 68.02%, respectively (Fig 1). Furthermore, the majority (96.07%) of the respondents have heard of HIV/AIDS. In this study, 74.55% of the women were aware HIV may be transferred during pregnancy, and 77.2% assumed that HIV can be transmitted during birth. Again, 79.27% of the women in the sample were aware that HIV might be transmitted during breastfeeding, and 76.77% believed that medications could be taken to prevent HIV transmission to the infant during pregnancy (Table 2).

**Table 2:** Knowledge of MTCT of HIV among reproductive women aged 15–49 years in high HIV prevalent countries of SSA, N = (97,130).

Knowledge of MTCT	Category	Frequency	Percentage
Ever heard of HIV/AIDS	No	3,814	3.93
	Yes	93,316	96.07
HIV is transmitted during Pregnancy	No	24,718	25.45
	Yes	72,412	74.55
HIV is transmitted during delivery	No	22,147	22.8
	Yes	74,983	77.2
HIV is transmitted by breastfeeding	No	20,135	20.73
	Yes	76,995	79.27
Drugs to avoid HIV transmission to the baby during pregnancy	No	22,561	23.23
	Yes	74,569	76.77
Overall good knowledge on MTCT	No	40,814	42.02
	Yes	56,316	57.98

### Factors associated with knowledge of MTCT of HIV among reproductive women aged 15–49 years in High HIV prevalent countries

The odds of knowledge of MTCT of HIV/AIDS among women aged, 25–34, 35 and above, 1.37 (95% CI = 1.32, 1.42), 2.46 (95% CI = 1.41, 1.52) times higher compared to those aged 15–24 years, respectively. The odds of knowledge of MTCT of HIV was 1.09 times (95% CI = 1.04,1.14) higher among women who lived in urban areas relative to those who lived in rural areas. Women who attended primary, secondary and higher education had 1.32 (95% CI = 1.26, 1.38), 1.65 (95% CI = 1.56, 1.74), and 1.72 (95% CI = 1.58,1.86) odds of knowledge of MTCT of HIV, respectively as compared to no education. Women who are exposed to mass media reported 1.12(95% CI = 1.08,1.16) odds of knowledge of MTCT of HIV compared to those who are not exposed. Category of women from households with middle, and rich wealth index level reported 1.11 (95% CI = 1.06,1.15) odds of knowledge of MTCT HIV compared to those who were from poor households. The likelihood of knowledge of MTCT HIV/AIDS was 1.70 (95% CI = 1.64, 1.76) times higher in women who talked about HIV mother-to-child transmission during antenatal visits compared to their counterparts. The odds of knowledge of MTCT of HIV/AIDS among women who are visited by field workers was 1.09(95% CI = 1.03,1.14) times higher compared to those who are not visited by field workers. Women who visited the health facility in the last 12 months reported 1.15(95% CI = 1.11, 1.18) odds of knowledge of MTCT HIV/AIDS compared to those who did not have a visit. Women who are ever tested for HIV reported 2.18 (95% CI = 2.10,2.27) odds of Knowledge of MTCT HIV/AIDS compared to those who did not have HIV test. Women currently working had 1.15(1.12,1.19) higher odds of knowledge of MTCT HIV/AIDS compared to those currently not working (Table 3).

**Table 3:** Factors associated with knowledge of MTCT of HIV among reproductive age women (15–49 years) in high HIV prevalent countries, N = (97,130).

Variables	Null model	Model I AOR (95% CI)	Model II AOR (95% CI)	Model III AOR (95% CI)
**Age of respondents**				
15–24		1		1
25–34		1.39 (1.34,1.44)		1.37(1.32, 1.42)*
≥35		1.53(1.47,1.59)		2.46(1.41,1.52) *
**Residence**				
Rural			1	1
Urban			1.30(1.26,1.34)	1.09(1.04,1.14)*
**Marital status**				
Married		0.99(0.96,1.02)		1.05(0.99,1.09)
Not married		1		
**Region**				
Equatorial Guinea			1	1
Lesotho			4.39 (4.10,4.69)	1.99(1.83,2.15)*
Malawi			5.49 (5.19,5.80)	2.64(2.48,2.83)*
Mozambique			3.61 (3.41,3.82)	2.54(2.38,2.70)*
Namibia			4.24 (3.98,4.51)	2.05(1.90,2.22)*
South Africa			4.79(4.48,5.12)	2.32(2.13,2.51)*
Zambia			3.25 (3.07,3.44)	1.48(1.38,1.58)*
Zimbabwe			5.83 (5.48,6.19)	2.74(2.54,2.95)*
**Media exposed**				
No		1		1
Yes		1.11(1.07,1.15)		1.12(1.08,1.16)*
**Educational status**				
No education		1		1
Primary education		1.68(1.61,1.76)		1.32(1.26, 1.38)*
Secondary education		2.05(1.95,2.15)		1.65(1.56, 1.74)*
Higher education		2.06(1.91,2.23)		1.72(1.58,1.86)*
**Wealth status**				
Poor		1		1
Middle		1.11(1.10,1.15)		1.11(1.06,1.15)*
Rich		1.13(1.09,1.17)		1.11(1.07, 1.16)*
**Community-level poverty**				
High			0.96(0.90,1.02)	0.97(0.91,1.03)
Low			1	1
**During antenatal visit talked about: HIV transmitted mother to child**				
No		1		1
Yes		1.73(1.67,1.79)		1.70(1.64, 1.76)*
**Visited by fieldworker**				
No		1		1
Yes		1.02(.97,1.06)		1.09(1.03,1.14)*
**Visited health facility**				
No		1		1
Yes		1.16(1.13,1.20)		1.15(1.11,1.18)*
**Ever been tested for HIV**				
No		1		1
Yes		2.43(2.35,2.52)		2.18 (2.10,2.27)*
**Respondent Currently working**				
No		1		1
Yes		1.11(1.07,1.14)		1.15(1.12,1.19)*
**Community level media exposure**				
High			0.99(0.93,1.05)	0.97(0.91,1.03)
Low			1	
**Community level women’s literacy**				
High			1.10(1.04,1.16)	1.03(0.98,1.09)
Low			1	1
**Distance to health facility**				
Big problem			1	1
Not big problem			1.09(1.05,1.12)	1.04(0.99,1.07)
Intercept	1.47(1.42,1.51)	0.24(0.23,0.25)	0.31(0.28,0.33)	0.14(0.12,0.15)
**Measure of variations**				
Log-likelihood	-65405.916	-60058.553	-62621.036	-59250.678
Deviance	130811.8	120117.1	125242.1	118501.4
Variance	0.124	0.096	0.097	0.095
ICC	3.64%	2.86%	2.89%	2.92%
PCV	Reference	22.58%	21.77%	23.39%

* p-value < 0.05

Null model-contains no explanatory variables; Model I-includes individual-level factors only; Model II-includes community-level factors only; Model III includes both individual-level and community-level factors, AOR-adjusted odds ratio, CI-confidence internal, ICC-intraclass correlation coefficient, PVC proportional change in variance

## Discussion

The purpose of this study was to assess reproductive-age women’s knowledge on MTCT of HIV/AIDS and associated factors. In our study, 57.98% of respondents were knowledgeable of HIV/AIDS MTCT and its prevention. This study was lower than a study conducted in Zambia 58.6% [27], Rwanda 65.1% [3], Zimbabwe 70.5% [19], Nigeria 78% [28], and in North West Ethiopia 88.5% [29], however this study report higher prevalence of knowledge of MTCT compared to study done in Ethiopia 34.9% [9], Mecha district Ethiopia (31.4%) [30], and in Tanzania 46% [31]. The lower prevalence of knowledge of MTCT in our study and the above study might be because some of these studies were done at institutional levels, where access to information, health education, and awareness of MTCT is likely to be increased. The higher prevalence of knowledge of MTCT in our study and the above study might be due to the difference in sample size, the study population, and the study period.

Age of the respondents, living in urban area, primary education and above, being exposed to mass media, having higher wealth household, talked about MTCT during ANC visit, visited by field worker, visit health facility in the last 12 months, respondent currently working, ever been tested for HIV were positively associated with women’s good knowledge of MTCT. Accordingly being in the age group of 25–34, 35 and above had higher odds of knowledge on MTCT of HIV/AIDS and its prevention as compared to women aged 15–24 years. This is in agreement with studies done in Tanzania [19] and SSA [32]. This might be linked to as age gets older, women’s experience to various maternal health services during each subsequent pregnancy also increased. This shows that special focus should be given for youth to enhance their awareness of HIV transmission and subsequently reduces MTCT.

The odds of knowledge of MTCT were higher among women who reside in urban areas when compared to rural dwellers. This is in line with different studies in Rwanda [3], in Tanzania [30], in Ethiopia [28, 29], and in south Africa [32]. The observed discrepancies might be related to the variations in the availability and quality of HIV-related information and other resources in different contexts. Evidence shows that living in rural areas has been shown to limit the utilization of HIV and EMTCT services [33, 34], which in return affects the awareness and knowledge of MTCT among rural dwellers. This implies that the need for targeted interventions, improved access to healthcare services, and efforts to address disparities in knowledge between urban and rural areas.

Women who had primary education and above had higher odds of knowledge of MTCT compared to those with no formal education. This is in agreement with different studies elsewhere [9, 30, 32]. This explains that education is helpful to increase the awareness and had an impact on health knowledge and behavior [33]. Women from the middle, and rich households had higher odds of Knowledge of MTCT than those who were from the poor households. This is in line with different studies [9, 31, 32]. The higher level of knowledge among women from higher socioeconomic class may be related to easier access to maternal health care services like PMTCT programmes. This showed that promoting education among women can be a key strategy in enhancing knowledge of MTCT, leading to improved prevention and management efforts.

In our study, women who exposed to mass media had higher odds of Knowledge of MTCT of HIV/AIDS when compared to their counterparts. This is consistent with studies done in SSA [32], and in Ethiopia [9]. Exposure to mass media has a greater influence on overall knowledge of mothers [34]. This means that in order to eliminate HIV MTCT, targeted MTCT and PMTCT messages must be delivered to them via various types of mass media.

Women who are being told about MTCT during ANC visit, visited by field workers, who visited health facilities in the last 12 months, and who had ever been tested for HIV had higher odds of knowledge of MTCT of HIV/AIDS. A similar finding was reported in Zimbabwe [19] and Rwanda [3]. This might be women who have been tested and counseled may be exposed to MTCT information during the antenatal visits, when they visit the health institution, and or when they are visited by the field workers. Furthermore, those who are tested for HIV may get post-test counselling which in return increases their knowledge of MTCT of HIV. This implies that there should be strategies that promote regular ANC visits, field worker visits, frequent health facility visits, and increased access to HIV testing.

Regarding the strengths, we used large data set from 8 high HIV prevalent countries DHS, which is representative across the country. In addition, we used a multilevel modeling approach to provide a more accurate conclusion that takes into the consideration of data nature of the survey (hierarchical). However, the study has limitations, One notable limitation is the reliance on secondary data from the DHS, which may not capture all the pertinent variables necessary for a comprehensive analysis of knowledge of MTCT. Furthermore, the cross-sectional design employed in this study precludes establishing causality.

## Conclusions

Overall, the prevalence of good knowledge of MTCT was low in high HIV/AIDS prevalent countries. Maternal age, primary education and above, exposed to media, having higher wealth status, talked about MTCT during ANC visits, being visited by a field worker, visited a health facility, currently working, living in the urban area, and ever been tested for HIV were positively associated with knowledge of MTCT. Health policies and programs need to prioritize the education of mothers and women of reproductive age regarding MTCT. Efforts should be made to encourage women to seek healthcare services, including ANC visits and HIV testing. Moreover, targeted communication programs should be developed and implemented to effectively enhance knowledge of MTCT of HIV/AIDS.

## Abbreviations

AIDS: Acquired immunodeficiency syndrome
AOR: Adjusted Odds Ratio
Human Immunodeficiency Virus (HIV): ICC: Intra-class Correlation Coefficient
MTCT: Mother-To-Child Transmission
WHO: World Health Organization
